# Detection of circulating tumor cells in blood of metastatic breast cancer patients using a combination of cytokeratin and EpCAM antibodies

**DOI:** 10.1186/1471-2407-12-206

**Published:** 2012-05-30

**Authors:** Ulrike Weissenstein, Agnes Schumann, Marcus Reif, Susanne Link, Ulrike D Toffol-Schmidt, Peter Heusser

**Affiliations:** 1 Society for Cancer Research, Hiscia Institute, Arlesheim, Switzerland; 2 Institute for Clinical Research, Berlin, Germany; 3 University Hospital Basel, Basel, Switzerland; 4 Center for Integrative Medicine, University of Witten/Herdecke, Witten, Germany

## Abstract

**Background:**

Circulating tumor cells (CTCs) are detectable in peripheral blood of metastatic breast cancer patients (MBC). In this paper we evaluate a new CTC separation method based on a combination of anti-EpCAM- and anti-cytokeratin magnetic cell separation with the aim to improve CTC detection with low target antigen densities.

**Methods:**

Blood samples of healthy donors spiked with breast cancer cell line HCC1937 were used to determine accuracy and precision of the method. 10 healthy subjects were examined to evaluate specificity. CTC counts in 59 patients with MBC were measured to evaluate the prognostic value on overall survival.

**Results:**

Regression analysis of numbers of recovered vs. spiked HCC1937 cells yielded a coefficient of determination of R^2^ = 0.957. The average percentage of cell recovery was 84%. The average within-run coefficient of variation for spiking of 185, 85 and 30 cells was 14%. For spiking of 10 cells the within-run CV was 30%. No CTCs were detected in blood of 10 healthy subjects examined.

A standard threshold of 5 CTC/7.5 ml blood as a cut-off point between risk groups led to a highly significant prognostic marker (p < 0.001). To assess the prognostic value of medium CTC levels we additionally considered a low (CTC-L: 0 CTC), a medium (CTC-M: 1–4 CTC) and a high risk group (CTC-H: ≥5 CTC). The effect of this CTC-LMH marker on overall survival was significant as well (p < 0.001). A log-ratio test performed to compare the model with 3 vs. the model with 2 risk groups rejected the model with 2 risk groups (p = 0.026). For CTC as a count variable, we propose an offset reciprocal transformation 1/(1 + x) for overall survival prediction (p < 0.001).

**Conclusions:**

We show that our CTC detection method is feasible and leads to accurate and reliable results. Our data suggest that a refined differentiation between patients with different CTC levels is reasonable.

## Background

In recent years results about the clinical relevance of circulating tumor cells (CTCs) in peripheral blood of patients with metastatic breast cancer (MBC) and other tumor types have accumulated [1-6]. Cristofanilli et al. demonstrated in 177 MBC patients that the number of CTCs before treatment is an independent predictor of progression-free and overall survival [2,3]. Elevated CTC levels during therapy further indicated subsequent rapid disease progression and mortality for MBC patients [4]. The correlation between CTC count and prognosis has been confirmed by several studies [5,6].

The main approaches to analyze CTCs derived from blood are immunological and PCR-based molecular assays. The frequency of tumor cells among normal blood cells is assumed to range from 10^−5^ to 10^−8^[7,8]. Because of this rareness, CTCs need to be enriched which is usually achieved by immunomagnetic separation. As standard markers for the immunocytochemical detection of CTCs the epithelial cell adhesion molecule (EpCAM) and cytokeratins (CK) are used. Several CTC assays used today are based on enrichment with anti-EpCAM antibodies and subsequent detection with anti-cytokeratin, for example the FDA approved CellSearch^TM^ system [9]. EpCAM as well as cytokeratin expressing cells can be found in peripheral blood of advanced cancer patients but are rare in healthy donors [1,10]. EpCAM is overexpressed 100- to 1000-fold in primary and metastatic breast cancer relative to normal breast cells [11]. Breast cancer cells of all grades typically express the epithelial cytokeratins CK7, CK8, CK18 and CK19 [12-14]. On the other hand expression of these antigens can vary widely in breast cancer cells and there is growing concern about consequences of this heterogeneity for CTC detection [15-18].

Deng et al. demonstrated the advantage of combining anti-EpCAM and anti-cytokeratin antibodies for CTC enrichment which compensates low or missing expression of either EpCAM or cytokeratins [15].

For the present study we modified a commercially available tumor cell enrichment and detection assay to combine anti-EpCAM and anti-cytokeratin for immunomagnetic CTC enrichment. Blood samples of healthy donors spiked with breast cancer cell line HCC1937 were used to determine accuracy, precision and specificity of the method. CTC levels of 59 MBC patients were measured and the prognostic significance regarding overall survival (OS) was examined. For survival analysis the conventional threshold of 5 CTCs/7.5 ml was used. But, only recently a discussion has started about the right way to use CTC measurements for risk assessments [19,20]. We particularly examine a further division of the 0–4 CTC group into a low risk group with 0 CTC and a medium risk group with 1–4 CTC and provide a sensitivity analysis with CTC as a count variable. The definition of a medium risk group, which was also used by Botteri et al. [19], was motivated by the assumption that already a single detected CTC reflects a higher probability for forthcoming death.

All analyses were performed according to the REMARK criteria [21,22].

## Methods

### Patients and blood collection

Our investigation was in compliance with the declaration of Helsinki. The study was accepted by the Ethics Committee of Basel, Switzerland (EKBB).

Blood samples were drawn after gathering informed consent from 10 healthy donors and from 59 MBC patients in the Lukas Clinic, Arlesheim. Patients with other tumor diagnoses before the breast cancer diagnosis were not considered eligible to avoid ambiguities over the onset of the disease. The blood samples were obtained between 08/2007 and 08/2008. Last update of survival information was made in April 2010. The sample size was dependent on the number of MBC blood samples send by doctors available within the time frame.

Blood was collected into Cell Save Preservative Tubes (Immunicon, Huntington Valley, PA, USA), maintained at room temperature until processing within a maximum of 96 hours.

### Sample preparation and analysis protocol for circulating tumor cell detection

CTC samples were prepared using the Carcinoma Cell Enrichment in combination with the Carcinoma Cell Detection Kit supplemented with CD326 (EpCAM) MicroBeads (Miltenyi Biotech GmbH, Bergisch-Gladbach, Germany).

The protocol was carried out according to manufacturer’s instructions. In brief, 7.5 ml anti-coagulated peripheral blood was centrifuged at 400xg for 35 minutes without brake and afterwards leukocyte enriched interphase (buffy coat) was carefully collected in a volume of 3.5 ml. Cells were permeabilized, fixed and incubated with FcR blocking reagent for 30 minutes. After the simultaneous incubation with anti-cytokeratin (specific for CK 7/8) - and anti-EpCAM-MicroBeads, anti-cytokeratin-alkaline phosphatase (specific for CK 7, 8, 18 and 19) was added. For the detection and quantification of unspecific binding due to Fc receptor binding or other protein-protein interactions mouse IgG1/IgG2a isotype controls (Miltenyi Biotech GmbH) were used at identical concentrations and staining conditions as the target primary antibodies.

Samples were applied to positive selection columns and placed in the magnetic field of a QuadroMACS^TM^ separation unit (Miltenyi Biotech GmbH). After washing with PBS, columns were detached from the cell separator and targeted cells were eluted.

Eluted target cells were spinned on Silane-Prep Slides (Sigma-Aldrich Logistik GmbH, Buchs, CH). Cell spots were dried and incubated with the freshly prepared substrate solution for the alkaline phosphatase color reaction. After mounting and drying, slides were now ready for microscopic visualization using a brightfield microscope.

Criteria for classification of a cell as circulating tumor cell were round or oval morphology, positive staining for cytokeratins and negative corresponding isotype control.

CTC measurements of all patients were performed blinded to the study endpoint overall survival.

### Accuracy, precision and specificity of circulating tumor cell detection

To estimate the accuracy and precision EpCAM^+^/CK^+^ breast cancer cell line HCC1937 (German Collection of Microorganisms and Cell Cultures, Braunschweig, Germany) was spiked into the blood of healthy donors in duplicates or triplicates with approximately 10, 30, 85 and 185 cells per 7.5 ml blood. Before spiking, the actual cell number of HCC1937 cell line was determined using BD TruCount tubes (BD Biosciences, San Jose, CA, USA), containing a known number of fluorescent beads and by running of samples on a flow cytometer (FACS Calibur, BD Biosciences, San Jose, CA). All 28 tubes were processed within 4 days after blood collection according to the sample preparation and analysis protocol for circulating tumor cell detection.

To investigate the specificity of the CTC detection method, 7.5 ml peripheral blood samples of 10 healthy volunteers (6 female and 4 male) were collected and analyzed according to the sample preparation and analysis protocol.

### Statistical analysis of CTC as a prognostic marker

Overall survival probability estimates for risk groups were visualized by Kaplan-Meier plots and compared by a standard log-rank test or a log-rank test for trend where appropriate [23]. Baseline blood collection was taken as the point of origin from which on survival was estimated. Cox proportional hazards model [23] was used to calculate p-values, hazard ratios (HR) and to compare models by a likelihood-ratio test. Median scores were used for a trend test in the Cox model. As a sensitivity analysis CTC were also included as a count variable in the Cox model using the supremum test for functional form [24] to choose an appropriate transformation. The model assumptions were checked by the supremum test for proportional hazards [24] and by a calculation of cumulative incidence rates [25] considering loss of patients due to unknown reason as a competing risk to survival. Fisher’s exact test was applied to check whether there were differences in early drop out reasons between risk groups. Demographic data, therapy information and other prognostic markers were traced retrospectively from patient's medical record and compared among risk groups by Fisher’s exact test and Cochrane-Armitage trend test. An assessment of the univariate prognostic value of each of the baseline parameters is performed and used as a basis for a multivariate Cox proportional hazards model. The final multivariate model was chosen by automatic variable selection. All calculations in this section were done with SAS® 9.2 for Windows. Statistical two-sided P-values <0.05 were considered significant. The statistical analysis of the recovery experiments was performed using MedCalc for Windows, version 9.3.8.0 (MedCalc Software, Mariakerke, Belgium).

## Results

### Accuracy, precision and specificity

The results of the recovery tests performed by spiking varying numbers of HCC1937 breast cancer cells into blood samples of healthy donors are summarized in Table 1. The relationship of the number of recovered vs. the number of spiked tumor cells was linear and regression analysis yielded a coefficient of determination of R^2^ = 0.957 (P < 0.001). The average percentage of HCC1937 cell recovery was 84% (95% CI = 78–90), the within-run coefficient of variation for the recovery rate lay between 12% and 30%. None of the 7.5 ml peripheral blood samples of the 10 healthy subjects analysed was found to have CTCs.

**Table 1:** Accuracy and Precision of the CTC detection method

**Expected CTC count**	**N**	**N**	**Observed CTC count**			**% Recovery**			
	donors	samples	Mean	SD	95% CI	Mean	SD	95% CI	% CV
10	2	6	8	2.4	6–10	80	24	55–105	30
30	3	8	25	3	22–27	82	10	74–90	12
85	3	8	76	14	65–88	90	16	76–104	18
185	2	6	151	18	131–171	82	10	71–92	12
TOTAL	5	28				84	15	78–90	18

Observed vs. expected numbers of CTCs and recovery measured in blood samples of 5 healthy donors spiked with defined numbers of HCC1937 tumor cells.SD = standard deviation, CI = confidence interval of the mean, CV = coefficient of variation (SD/mean).

### Prognostic value of CTCs detected in peripheral blood samples of MBC patients

CTC levels of 59 MBC patients were measured to assess their prognostic value for overall survival prediction. 20 patients had no CTCs, 15 patients had 1-4 CTCs and 24 had ≥5 CTCs/7.5 ml blood. During the follow-up period 26 of the 59 patients died. Patients were followed as long as they were accessible to the clinician (17 patients) or until the end of the overall study (14 patients); 2 patients were lost because a study clinician left the clinic. The median follow-up time for the patients still alive at the end of the study was 85 weeks (range 7-134 weeks).

Following the suggestion in [2] using the Veridex Cell Search system, we could confirm that differentiating between patients with ≥5 CTC vs. <5 CTC led to a very strong risk marker for overall survival (p = 0.00006 log-rank test). Median overall survival of the ≥5 CTC group was 60 weeks compared to >100 weeks in the <5 CTC group; quartile survival times were 22 vs. 85 weeks, respectively. The hazard ratio HR of ≥5 CTC vs. <5 CTC estimated by Cox model was 4.79 (p = 0.0002, 95% CI = [2.09,11.0]).

**Figure 1:**
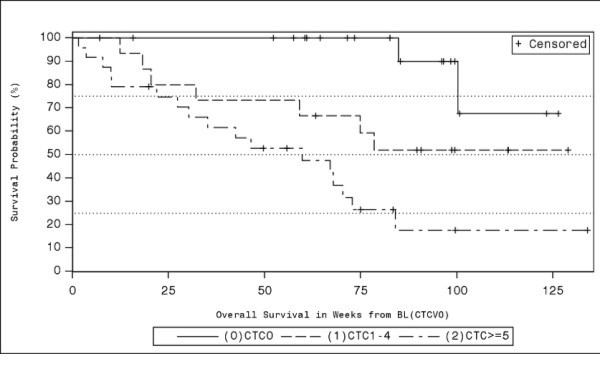
Kaplan-Meier plot estimating overall survival for 3 risk groups (0 CTC, 1–4 CTC, ≥5 CTC).

To further investigate the relationship between CTC number and prognosis we defined 3 risk groups, a low (CTC-L: 0 CTC), a medium (CTC-M: 1-4 CTC) and a high risk group (CTC-H: ≥5 CTC). The 3-valued marker CTC-LMH proved to be significant as well (p = 0.00002 log-rank test for trend, p = 0.0001 trend test with Cox model). The differentiation between the low and the medium risk as well as between the medium and the high risk groups were both significant in the Cox model (CTC-M vs. CTC-L: HR = 4.94, 95% CI = [1.02,23.8], p = 0.046; CTC-H vs. CTC-M: HR = 2.55, 95% CI = [1.04,6.3], p = 0.042). A log-ratio test performed to compare the model with 3 vs. the model with 2 risk groups rejected the model with 2 risk groups (p = 0.026). The median survival was shortest in the high risk group (60 weeks), followed by the medium (>78 weeks) and low risk group (>100 weeks). Corresponding quartile survival times were 22, 32 and 100 weeks (Table 2). Kaplan-Meier estimates (KM) of survival probabilities for each risk group are shown in Figure 1. There were no major differences in early drop out reasons between risk groups (p = 0.63) and the cumulative incidence curves were similar to 1-KM estimates.

**Table 2:** Survival data of 59 patients stratified into 3 risk groups according to baseline CTC counts

Risk Group	Death N (%)	Censored N (%)	Quartile Survival [weeks]	Median Survival [weeks]
CTC-L (0 CTC) N = 20	2 (10.0)	18 (90.0)	100 95%CI = [85,+oo]	>100 95%CI = [85,+oo]
CTC-M (1-4 CTC) N = 15	7 (46.7)	8 (53.3)	32 95%CI = [12,78]	>78 95%CI = [20,+oo]
CTC-H (≥5 CTC) N = 24	17 (70.8)	7 (29.2)	22 95%CI = [2,42]	60 95%CI = [27,73]
TOTAL N = 59	26 (44.1)	33 (55.9)	46 95%CI = [20,70]	100 95%CI = [70,+oo]

Quartile and median survival times are given together with confidence intervals (CI). N is the number of patients in the respective group.

As sensitivity analysis we included CTC as a count variable into the Cox model without dichotomization. Due to the positively skewed CTC distribution, the significance of CTC as a survival predictor is lost when CTC counts are used without transformation (p = 0.056). Considering the supremum test for functional form, we propose an ‘offset reciprocal’ transformation y = 1/(1 + x) (p = 0.0002, HR 0.063 for a change of 1 on transformed scale). On our data, the log(x + 1)-transformation used by Botteri et al. [19] did not pass the supremum test for functional form. Using the ‘offset reciprocal model’ for successive deterioration from 0 to 5 CTC, we get hazard ratios of 3.98 (1 vs. 0 CTC), 1.58 (2 vs. 1 CTC), 1.26 (3 vs. 2 CTC), 1.15 (4 vs. 3 CTC) and 1.10 (5 vs. 4 CTC). This sequence rapidly converges to 1 indicating that the definition of risk groups is more effective in the low level range.

From a practical point of view, it is helpful to define risk groups and describe them by mean characteristics and respective course of disease. We compared them in regard to demographic data, treatment and several prognostic markers and evaluated the prognostic value of each particular parameter for overall survival prediction (Table 3). Among the predominantly female breast cancer patients there was one male patient who belonged to the high risk group. Two patients in the low risk group suffered from a secondary tumor (Ovarian/Uterus) diagnosed after the breast cancer diagnosis. The mean age at baseline was 57.1 ± 10 years (CTC-L: 57.6 ± 9, CTC-M: 60.2 ± 10, CTC-H:54.8 ± 11). The fraction of patients with positive lymph nodes at time of BC diagnosis increased from the low to the high risk group (p = 0.03). There was a significant difference in the fraction of patients with bone metastases which was highest in the high risk group (p = 0.02). The high risk group had significantly more patients with an elevated level for the tumor marker Carbohydrate Antigen 15-3 (CA 15-3) (p = 0.02). The inflammatory marker C-reactive protein (CRP) displayed a significant positive trend (p = 0.03).

**Table 3:** Comparison of risk groups on demographic or treatment characteristics and other prognostic markers

**Parameter**	**Low Risk (CTC-L) CTC = 0**		**Medium Risk (CTC-M)CTC1-4**		**High Risk (CTC-H) CTC > =5**		**TOTAL**		**Association with LMH p-Value**	**Association with OS p-value (HR)**
	N/N_G_	%	N/N_G_	%	N/N_G_	%	N/N_G_	%		
Patients with age at TSE >50	16/20	80	13/15	87	14/24	58	43/59	73	n.s.	n.s.
Patients with age at BC diagnosis >50	9/20	45	10/15	67	11/24	46	30/59	51	n.s.	n.s.
T3/T4 at BC diagnosis	2/16	12.5	4/10	40	5/20	25	11/46	24	n.s.	<0.01 (HR = 5.1)
N0 at BC diagnosis (NX = missing value)	7/16	44	2/10	20	2/19	10.5	11/45	24	0.03 (CAX)	0.001
ER/PR at least one positive	15/19	79	8/13	61.5	18/22	82	41/54	76	n.s.	0.02 (HR = 0.37)
HER2 overexpressed	4/14	29	2/11	18	4/21	19	10/46	22	n.s.	n.s.
CA 15–3 above normal value of 31.3 (≤ 30 days before TSE)	9/13	69	6/9	67	18/18	100	33/40	85	0.02 (FSHX) 0.03 (CAX)	n.s.
CRP above normal value of 5 (≤ 30 days before TSE)	3/12	25	5/8	62.5	13/19	68	21/39	54	0.03 (CAX)	n.s.
Visceral metastases	8/20	40	7/14	50	10/23	43.5	25/57	44	n.s.	n.s.
Nonvisceral metastases	20/20	100	12/14	86	22/23	96	54/57	95	n.s.	n.s
Bone metastases	14/20	70	6/14	43	20/23	87	40/57	70	0.02 (FSHX)	n.s.
No. of metastatic sites > =2	9/20	45	8/14	57	11/23	48	28/57	49	n.s.	0.02 (HR = 2.8)
Surgery of primary tumor	17/20	85	13/15	87	19/24	79	49/59	83	n.s.	n.s.
Radiation therapy	14/19	74	9/13	69	16/22	73	39/54	72	n.s.	n.s.
Chemotherapy	14/18	78	10/14	71	17/21	81	41/53	77	n.s.	n.s.
Mistletoe therapy	20/20	100	15/15	100	24/24	100	59/59	100	-	-
Anti-hormone therapy	16/19	84	8/13	61.5	19/23	83	43/55	78	n.s.	0.01 (HR = 0.34)
Bisphosponate therapy	11/17	65	6/14	43	17/19	89.5	34/50	68	0.03 (FSHX)	n.s.
Patients with ≥2 known therapy lines	15/20	75	6/15	40	14/24	58	35/59	59	n.s.	n.s.

N is the number of patients, N_G_ denotes the number of patients with non-missing values in the respective group. P-values are given if significant (p < 0.05), n.s. denotes “not significant”. LMH denotes the prognostic marker which differentiates between low (L), medium (M) and high (H) CTC levels. Risk groups are compared by the exact Cochrane Armitage trend test (CAX) or Fishers Exact Test (FSHX). Time of study entry (TSE) is defined as time of baseline blood draw. For each parameter in the table the univariate prognostic value for overall survival (OS) prediction is given in the last column. P-Values and Hazard Rates (HR) in this column are computed by Cox Proportional Hazard Regression; except for “N0 at BC diagnosis” the logrank test was used because of no events (deaths) in the reference group.

The significance of CTC-LMH is also supported by multivariate models (Model 1: CTC-LMH (p < 0.001), ER/PR at least one positive (p = 0.04), age at BC diagnosis >50 years (p = 0.03) and T3/T4 at BC diagnosis (p = 0.002); Model 2 additionally stratified by N0 at BC diagnosis^a^: CTC-LMH (p = 0.001), ER/PR at least one positive (p = 0.005), age at BC diagnosis >50 (p = 0.03) and T3/T4 at BC diagnosis (p = 0.08).

## Discussion

Most immunocytochemical CTC detection technologies are based on a separation of CTCs from normal blood cells with EpCAM antibodies. The exact biological function of EpCAM is not fully understood and remains controversial. In some publications EpCAM is argued to act as an intercellular adhesion molecule, and loss of EpCAM expression therefore reduces cell-cell adhesion, thereby promoting dissemination of tumor cells [26]. In contrast, Osta et al. [11] report that silencing EpCAM gene expression in vitro decreases the proliferation, migration and invasion potential of breast cancer cell line MD-MB-231. EpCAM might also have a dual role as cell adhesion molecule and receptor involved in the regulation of gene transcription and cell proliferation [27].

Our CTC detection method is based on a commercially available Carcinoma Cell Enrichment and Detection Kit which uses CK7/CK8 MicroBeads supplemented by EpCAM-MicroBeads. In former, very limited experiments with the commercially available kit based on CK-MicroBeads alone, a considerable amount of CTCs missed the magnetic attachment and was found in the flow through cell fraction (mean efficiency 44%). The objective of the CK/EpCAM combination was to improve detection rates for CTCs with low target antigen densities. Because the expression of EpCAM and cytokeratins in epithelial tumor cells can vary *per se*, phenotypes with low or missing expression of these epithelial specific antigens exist [28-30]. Based on this heterogeneous expression pattern CTCs can be classified into EpCAM^+^/CK^-/low^, EpCAM^-/low^/CK^+^ and EpCAM^+^/CK^+^[15,16,31]. E.g., Rao et al. investigated the expression of EpCAM and its co-expression with the epithelial cell specific markers CK 8/18 or CK 19 and Muc-1 in carcinoma cells present in blood [16]. They found CK^-^ CTCs in about 23% of blood samples of 30 carcinoma patients but only 0.2% EpCAM^-^ CTCs. Yet, they demonstrated that the efficiency of immunomagnetic recovery with anti-EpCAM coated ferrofluids is rapidly declining with decreasing EpCAM antigen density. Furthermore, Mego et al. [17] give preliminary data on breast cancer patients where the loss of epithelial antigen on CTCs due to epithelial mesenchymal transition might be responsible for partially missed CTCs by the CellSearch System. Deng et al. processed blood samples from 49 metastatic breast cancer patients with the CellSearch™ system and in parallel by a combined anti-EpCAM- and anti-cytokeratin magnetic cells separation method, comparable to our method. They obtained a significantly higher CTC positive rate (49% vs. 29%) and a larger dynamic CTC detected range (1 to 571 vs. 1 to 270) than that of the CellSearch™ system [15]. Another recent study indicated that the so-called normal genotype of invasive breast cancer, which accounts for approximately 10% of all cases, is typically negative for EpCAM expression and may thus be a cause of false-negative CTC determinations [18].

To estimate the accuracy, linearity and precision of our CTC detection method, the breast cancer cell line HCC1937 was spiked into the blood of healthy donors. The average percentage of cell recovery was 84% which is well within the recovery range of 60 to 85% of comparable published CTC detection methods [1,15,32-34]. The within-run coefficients of variation (CV) for the spiking of cells (12, 18, 12 and 30% for 185, 85, 30 and 10 cells, respectively) were comparable to CVs detected by Allard et al. [1] (8.2, 15.4, 22 and 47% for 319, 58, 18 and 4 cells, respectively). The variation of recovered cell numbers can partially be explained by the difficulty to accurately and reproducibly spike low numbers of cells that tend to form adherent clusters. Although a large volume of the buffy coat was collected, we can not entirely exclude the loss of cells due to this step.

For our assay, criteria for classification as circulating tumor cells were round or oval morphology, positive staining for cytokeratin and negative corresponding isotype control. Because the standard kit does not include CD45 labeling to specify leukocytes and our method is a nonfluorescent immunocytochemical method that allows the chromogenic detection of only one marker simultaneously, the unambiguous characterization of CTCs as nucleated, CD45 negative cells was not possible. However, after optimizing our assay in proof-of-concept experiments we were able to reduce the non-specific binding of antibodies to 0%, fulfilling the criteria for the evaluation of rare immunocytochemically identifiable cancer cells, defined by the European ISHAGE Working Group 1999 [35].

Using blood samples of 59 MBC patients and a standard threshold of 5 CTCs to define 2 risk groups, we can confirm the high prognostic value of this dichotomization. But, we hypothesized that there is more information in low CTC levels than generally recognized and defined a medium risk group CTC-M between the low risk group CTC-L with 0 CTC and the high risk group CTC-H with ≥5 CTC. The 3-valued marker CTC-LMH proved to be significant as well and in a statistical comparison of the model with 3 vs. 2 risk groups, the model with 2 risk groups was rejected. It is theoretically possible that the medium survival risk of patients with medium CTC-level is only because this group contains more patients with events falsely classified as CTC than the high risk group. Tibbe et al. [36] used a theoretical model with equal death risk for all patients with true CTC in their blood circulation (regardless of CTC level) and got rather extreme (unrealistic) results. The only analyses with more than 2 risk groups we know are described in [6,19] where 1, 5 and 20 were used as cut-off values. In their study the corresponding survival curves also exhibited low, medium and high risk corresponding to CTC-level, therefore suggesting a continuous nature of association although after approximately 5 CTCs the increase rate lessened. Fehm et al. and Botteri et al. recently discussed the relationship between CTC as a count variable and clinical outcome [19,20]. But yet, to our knowledge there has not been a broad debate on the existence of more than 2 risk groups or on the kind of relationship of CTC-level as a count variable to survival. We believe that such a debate would help to assess the clinical impact of medium changes in CTC level and it could provide a basis for a better understanding of disease stages or activity.

It is a limitation of our study that due to the retrospective collection of baseline data from medical records a considerable proportion of this data is missing. But, the analysis of demographic and baseline data in our study confirms known relationships of CTCs in peripheral blood to other variables. Similar to our study Müller et al. [37] report about an elevation of CA 15-3 in patients with detectable CTCs, probably a reflection of metastatic tumor burden. According to Nole et al. [6] the presence of ≥5 CTCs at baseline was associated with a higher number of positive lymph nodes, with an elevated CA 15-3 value, with non-overexpressing Her2/neu tumors and with the presence of bone metastases at baseline. The association of high CTC counts with the presence of bone metastases was shown in patients with different metastatic cancers [6,38-42]. As most patients with bone metastases were found in the high risk group it is coherent that this group most frequently receives Bisphosphonate treatment which is a standard treatment of malignant bone diseases. In our study patients with measurable CTCs had also elevated systemic CRP levels. Elevated biomarkers of inflammation like CRP are associated with reduced survival among breast cancer patients [43] which suggest that CRP may be related to tumor burden or progression and that chronic inflammation promotes mammary tumor development.

## Conclusions

We could show that our immunocytochemical CTC detection method allows the accurate precise and specific quantification of circulating epithelial tumor cells in a quality comparable to methods described in the current literature. It was proven that the number of CTCs detected by our method directly relates to survival of MBC patients and is associated to various clinical factors. We would like to contribute to a discussion about existence and proper definition of risk groups or the relationship of CTC-level to survival.

## Endnote

^a^ The stratification for N0 was chosen because otherwise the parameter showed significant deviations from the proportional hazards assumption (p < 0.0001) and there were no events (deaths) in the N0 group.

## Competing interests

The authors declare that they have no competing interests.

## Authors’ contributions

UW and PH made substantial contributions to the conception and design of the study, UW, SL and UT adapted the assay and performed analyses of blood samples, AS performed statistical analysis, UW, AS and MR interpreted data and drafted the manuscript. All authors read and approved the final manuscript.

## Pre-publication history

The pre-publication history for this paper can be accessed here:

http://www.biomedcentral.com/1471-2407/12/206/prepub
